# Anesthesia and Pain Management for Scoliosis Surgery: A Narrative Review

**DOI:** 10.1097/BSD.0000000000001758

**Published:** 2025-01-03

**Authors:** Małgorzata Reysner, Grzegorz Kowalski, Alicja Geisler-Wojciechowska, Tomasz Resyner, Katarzyna Wieczorowska-Tobis

**Affiliations:** Chair and Department of Palliative Medicine, University of Medical Sciences, Poznań, Poland

**Keywords:** somatosensory-evoked potentials, general anesthesia, regional anesthesia, multimodal anesthesia, dexamethasone, ketamine, dexmedetomidine, intrathecal morphine, epidural anesthesia

## Abstract

**Study Design:**

This was a narrative review.

**Objective:**

The objective of this review was to summarize the current evidence and knowledge gaps regarding anesthesia and pain management for scoliosis surgery, including multimodal analgesia, and identify the best anesthetic approach to scoliosis surgery that ensures patient safety and pain relief even in the postoperative period, with minimal influence on SSEP monitoring.

**Summary of Background Data:**

Spinal surgeries and fusions for scoliosis are associated with high pain levels. Inadequate analgesia can cause patient dissatisfaction, delay recovery, and increase the risk of chronic pain. Despite serious side effects, opioids are the mainstay of pain medication after scoliosis surgery. However, increasing emphasis on minimizing opioids and accelerating recovery has increased the adoption of multimodal analgesic therapy.

**Materials and Methods:**

The literature review was performed on standards of care, a pain management protocol, current therapeutic options, and innovative treatment options for patients undergoing scoliosis surgery. The literature was reviewed through 4 electronic databases: PubMed, Cochrane Library, Google Scholar, and Embase.

**Results:**

The initial search yielded 994 articles. Forty-seven relevant articles were selected based on relevance, recentness, search quality, and citations. Ten studies described the influence of different methods of anesthesia on neuromonitoring. Twenty-one researchers studied the effect of analgesics and coanalgesics on pain relief protocol. Nine studies treated regional anesthesia and its influence on pain management.

**Conclusions:**

The most suitable anesthetic approach that does not disturb the neuromonitoring is obtained by combining total intravenous anesthesia (TIVA) with remifentanil and propofol with regional anesthesia, particularly erector spinae plane block (ESPB), as a part of a multimodal analgesia protocol.

**Level of Evidence:**

Level II.

Posterior spinal fusion for pediatric idiopathic scoliosis is a highly invasive procedure. Surgically requires very high perioperative analgesia and may be associated with PPP (persistent postsurgical pain). Inadequate analgesia can delay achieving recovery goals such as oral feeding and movement, leading to patient/family dissatisfaction and increasing the risk of chronic postoperative pain. Traditionally opioids have been central to pain management after scoliosis surgery, despite several side effects,^1,2^ like vomiting, pruritus, constipation, and respiratory depression.

The anesthesia regimen for spine surgery facilitates neurophysiological monitoring of spinal cord integrity by SSEP (somatosensory-evoked potentials).^3^ Anesthesia significantly impacts SSEP monitoring. Therefore, choosing the proper anesthetic and analgesic drug may be crucial in monitoring spinal cord integrity.

Multimodal analgesia is considered the optimal method for perioperative pain management by targeting multiple pain pathways. Multimodal analgesia includes preoperative, intraoperative, and postoperative analgesic therapies aimed at combining multiple analgesic therapies to maximize analgesic efficacy while minimizing unwanted side effects. Multimodal analgesia includes pharmacological agents, axial anesthesia, and PNBs (peripheral nerve blocks).^4^ Compared with monotherapy, multimodal analgesia provides superior postoperative pain relief, promotes recovery, and reduces opioid use and associated side effects. As a result, multimodal analgesia is the gold standard for perioperative pain management in patients undergoing spine surgery.

This review aims to identify the best anesthetic approach to scoliosis surgery that ensures patient safety and pain relief even in the postoperative period, with minimal influence on SSEP monitoring.

## MATERIALS AND METHODS

**FIGURE 1:**
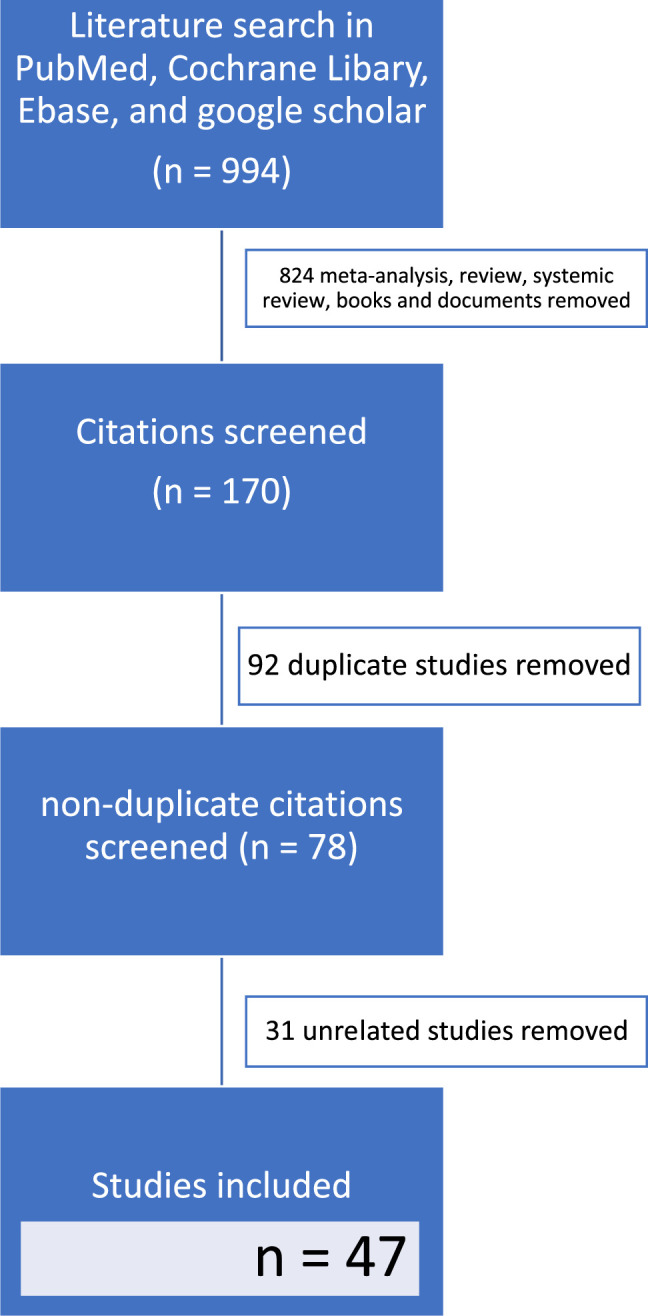
Flow chart of the search for published reports.

The literature was reviewed through 4 electronic databases: PubMed, Cochrane Library, Embase, and Google Scholar. We have limited the Google scholar search to the first 200 hits. This search was performed in February 2023. We evaluated studies published between 2017 and 2023 using the following search terms: “scoliosis” (title), “anesthesia” (title), “pain” (title), and “spine surgery” (title). The titles, abstracts, and full texts of published studies were screened. This entire process is shown in Figure 1.

Excluded literature spanned research involving reviews, meta-analyses, books, and protocols. M.D. and G.K. holistically assessed article inclusion, with all discordance reviewed for final inclusion by the senior author, K.W.T. Results from the included articles have been summarized as a narrative review to identify the most critical aspects of the known and unknown in this literature.

## RESULTS

The initial search yielded 994 articles. Forty-seven relevant articles were selected based on relevance, timeliness, search quality, and citations. The results are presented in 3 tables to facilitate the analysis of the collected material. Table 1 includes 10 studies describing the influence of different methods of anesthesia on neuromonitoring. Table 2 consists of 21 researchers studying analgesics and coanalgesics’ effect on pain management. Finally, Table 3 includes 9 studies that treated regional anesthesia and its influence on pain management.

**TABLE 1:** The Impact of Anesthesia on SSEP

Year	References	Type of study	Sample size	Method	Results
2023	Jiang^5^	Randomized double-blinded	90	Dexmedetomidine vs. placebo	Low-dose Dex does not affect the SEPs and tcMEPs (*P*<0.05)
2022	Liu^6^	Randomized double-blinded	160	Dexmedetomidine bolus vs. dexmedetomidine infusion vs. placebo	A bolus dose of dexmedetomidine increases the latency of SSEP (*P*<0.05) and reduces the amplitude of SSEP and MEP (*P*<0.05)
2022	Andleeb^7^	Randomized double-blinded	90	Dexmedetomidine vs. ketamine vs. placebo	A subanesthetic dose of ketamine causes improvement in amplitudes without affecting the latency (*P*<0.05)
2021	Furutani^8^	Randomized double-blinded	20	1 mg/kg/h Ketamine vs. placebo	Bolus dose of ketamine reduces MEP amplitudes (*P*<0.05)
2021	Panse^9^	Randomized	20	Dexmedetomidine vs. fentanyl	Dexmedetomidine and fentanyl does not influence SSEP (*P*=0.37)
2020	Parimal^10^	Randomized comparative study	40	Dexmedetomidine vs. Sevoflurane	Sevoflurane decreases the amplitude and increases the latency of SSEP (*P*<0.05)
2020	Sachdev^11^	Randomized double-blinded	60	Ketamine vs. dexmedetomidine	MAP was higher with Ketamine (*P*<0.05)
2020	Mishra^12^	Randomized double-blinded	30	Desflurane-dexmedetomidine vs. propofol-dexmedetomidine	No difference in MEP amplitude (*P*>0.05)
2019	Aggarwa^13^	Randomized double-blinded	60	Dexmedetomidine vs. midazolam	Decrease in MEP (6% vs. 33%; *P*=0.01)
2018	Hasan^14^	Randomized	64	Desflurane/remifentanil vs. TIVA	Desflurane had a greater reduction in amplitude (*P*=0.004) and an increase in latency (*P*=0.002)

**TABLE 2:** Analgesics and Coanalgesics in pain management

Year	References	Type of study	Sample size	Method	Results
2021	Vivekanandaswamy^15^	Randomized	63	Morphine vs. dexmedetomidine	NRS 3.1 vs. 2.7; *P*=0.07 Breakthrough analgesia regimens (0.78 vs. 0.49; *P*=0.17)
2021	Hong^16^	Retrospective	78	Dexmedetomidine vs. placebo	Lower PACU pain scores (*P*<0.05)
2020	Ricciardelli^17^	Randomized double-blinded	50	Ketamine vs. placebo	Reduction in opioid consumption (*P*=0.042) Adjustment in postoperative pain scores (*P*<0.001)
2017	Perello^18^	Randomized	48	Ketamine vs. placebo	Total cumulative opioid requirements (2.72 vs. 3.13; *P*=0.29)
2021	Julien-Marsollier^19^	Randomized double-blinded	33	Opioid-reduced anesthesia (ORA) vs. opioid-based anesthesia	Morphine consumption (0.8 mg/kg vs. 1.1 mg/kg; *P*=0.02) Decreased neuropathic pain with ORA |(*P*=0.02)
2020	Helenius^20^	Randomized	63	Pregabalin vs. placebo	opioid consumption (1.59​​​​​​ vs. 1.45 mg/kg; *P*=0.433)
2023	Zhang^21^	Retrospective	682	Gabapentin vs. placebo	14% change in opioid use (*P*<0.001)
2020	Anderson^22^	Randomized	50	Gabapentin vs. placebo	Lower pain scores (*P*<0.1)
2022	Kamel^23^	Randomized double-blinded		MgSO4 bolus vs. infusion	Postoperative requirement for analgesics 6.6% vs. 10%
2020	Dehkordy^24^	Randomized double-blinded	80	Magnesium vs. saline	Lower morphine consumption (*P*<0.005) Lower VAS score (*P*<0.005)
2021	Venkatraman^25^	Randomized double-blinded	100	morphine vs. fentanyl	Morphine is more effective in postoperative pain management
2021	Aoki^26^	Retrospective	142	Remifentanil vs. fentanyl	No correlation between total intraoperative remifentanil dosage and fentanyl consumption in the ICU (*P*=0.13)
2019	Kars^27^	Retrospective	62	Remifentanil vs. fentanyl	Postoperative narcotic use (0.5 vs. 0.8; *P*=0.0002) VAS score (2.1 vs. 3.7; *P*=0.05)
2021	Lo^28^	Retrospective	78	Association between remifentanil dose and opioid consumption after surgery	Opioid dose (*P*=0.588)
2021	Shaw^29^	Retrospective	26	Methadone vs. placebo	VAS (*P*=0.572)
2020	Ye^30^	Randomized double-blinded	122	Methadone vs. morphine	Lower morphine consumption (*P*<0.001) Higher pain scores on day 1 (*P*=0.01)
2020	Tams^31^	Retrospective	39	Methadone vs. placebo	NRS (3.4 vs. 4.8; *P*=0.03) Morphine milligrams equivalents (10 vs. 41 mg; *P*<0.01)
2018	Martin^32^	Randomized double-blinded	60	Methadone vs. placebo vs. magnesium	Total opioid requirement (0.26 vs. 0.34; *P*=0.035)
2020	Fletcher^33^	Retrospective	65	Dexamethasone vs. placebo	Morphine milligrams equivalents (49.5 vs. 82.0 mg; *P*<0.001)
2019	Wakamiya^34^	Randomized double-blinded	100	Dexamethasone vs. placebo	PONV (62% vs. 95%; *P*=0.02) Lower VAS score (*P*<0.01)
2018	Pieters^1^	Randomized	84	Low to high-dose naloxone infusion	No difference in VAS score and morphine equivalents

**TABLE 3:** Regional Anesthesia in Pain Management

Year	References	Type of study	Sample size	method	Results
2022	Poe-Kochert^35^	Retrospective	97	Intrathecal morphine vs. placebo	The first dose of opioids (16.8 vs. 5.5; *P*=0.001)
2022	Feltz^36^	Retrospective	105	Intrathecal morphine vs. placebo	Analgesic equivalence (388.4 vs. 167.2; *P*<0.0001) Number of midnights in hospital (6.0 vs. 4.2; *P*<0.0001)
2022	Hasan^37^	Retrospective	363	Intrathecal morphine vs. patient-controlled analgesia	Length of stay (5 vs. 3; *P*<0.0001)
2021	Li^38^	Randomized	50	Intrathecal morphine vs. Intrathecal morphine with oral gabapentin	Oxycodone consumption (0.798 vs. 1.036; *P*<0.015) Pain score (2.4 vs. 3.7; *P*=0.026)
2020	Thompson^39^	Retrospective	986	Intrathecal morphine vs. morphine	The first dose of opioids (10.6 vs. 2.3; *P*=0.001)
2020	Ina^40^	Retrospective	42	Intrathecal morphine vs. placebo	VAS (0.2 vs. 2.1; *P*<0.001)
2017	Erdogan^41^	Randomized	47	Patient-controlled intermittent bolus epidural analgesia vs. patient-controlled continuous epidural analgesia	No difference in the VAS score Pruritus (4 vs. 8; *P*=0.019) Morphine usage (5 vs. 12.5; *P*<0.0001)
2017	Cohen^42^	Randomized	71	Extended-release epidural morphine vs. intrathecal morphine	Total morphine consumption (42.2 vs. 34; *P*=0.27)
2020	Singh^43^	Randomized double-blinded	40	ESPB vs. placebo	Cumulative morphine requirement (1.4 vs. 7.2; *P*<0.001) The first dose of rescue analgesia (5.8 vs. 2.42; *P*=0.003)

## DISCUSSION

### Anesthesia and Neuromonitoring

The anesthesia regimen for spine surgery facilitates neurophysiological monitoring of spinal cord integrity by SSEP, routinely used to monitor the integrity of the spinal cord during scoliosis surgery. Intraoperative changes in SSEP can be caused by surgical injury or spinal cord ischemia.^5^ In addition, the SEESP signal can be affected by certain physiological variables, such as blood gases (PaCO_2_ and PaO_2_), hemoglobin concentration, blood pressure, and temperature. Also, anesthesia has a significant impact on SSEP monitoring. Increasing the anesthetic concentrations suppresses the amplitude of the evoked potentials (EP) and lengthens the latency period. This effect is time and dose dependent and reversible. Therefore, anesthesiologists play an essential role in selecting anesthesia techniques and agents to maintain the quality of SSEP and avoid misunderstandings. Table 1 includes a summary of the literature describing the influence of the type of anesthesia on neuromonitoring during scoliosis surgery.

Most studies have shown intravenous anesthesia superior to inhaled anesthesia for SSEP monitoring in spine surgery.^1,6,7^ Inhaled anesthetic impaired alpha motor neuron excitability and thus significantly reduced transcranial MEP.^8–11^ Several studies over the years have shown that propofol intravenous anesthesia (TIVA) with remifentanil caused less suppression in SSEP^11,12^ at an equivalent depth of anesthesia, in contrast to inhalation anesthesia, especially with nitrous oxide.^1,2,13^ Therefore, TIVA with remifentanil and propofol has been recommended for spine surgery for many years.^1,2,13^

### Analgesics and Coanalgesics in Pain Management

Postoperative pain remains a significant challenge after scoliosis surgery. Tissue injury and high opioid requirements following posterior spinal fusion surgery produce central sensitization, which can, in turn, be associated with hyperalgesia and chronic pain.^14^ Therefore, monotherapy alone cannot relieve postoperative pain after scoliosis surgery. Currently, multimodal analgesia is considered the optimal method for perioperative pain management for correcting scoliosis by targeting numerous pain pathways. Table 2 summarizes the literature describing the influence of analgesics and adjuvants on pain management in patients undergoing scoliosis surgery.

Acute opioid tolerance and/or hyperalgesia resulting from remifentanil-based anesthesia may involve activation of *N*-methyl-d-aspartate systems. Therefore, some researchers hypothesized that low-dose intraoperative infusion of the *N*-methyl-d-aspartate antagonist ketamine^15^ suppresses the development of tolerance and thereby decreases postoperative morphine consumption in patients receiving remifentanil-based anesthesia for scoliosis surgery. Whereas most general anesthetics depress motor-evoked potentials (MEPs) amplitude, the effect of ketamine has been dose dependent. Furutani et al^16^ showed that infusion of Ketamine ≥1.0 mg/kg suppressed the MEP amplitude. Subanesthetic doses of ketamine caused improvement in amplitudes without affecting the latency.^17,18^ However, Perelló et al^19^ did not support the routine of combining prolonged subanesthetic ketamine dose with opioids due to no differences in morphine consumption between ketamine and placebo.

Also, magnesium produces voltage-gated noncompetitive blockade at the NMDA receptor, preventing glutamate binding. Although magnesium is an inexpensive agent with a wide therapeutic threshold studied during orthopedic spine surgery, it has been shown to reduce postoperative opioid requirements, and intraoperative anesthetic needs.^20,21^ However, Martin et al^22^ noted no difference in pain scores and opioid consumption when magnesium was added to remifentanil.

Another drug routinely used in opioid-sparing multimodal anesthesia protocols is dexmedetomidine. Dexmedetomidine, an α-2 adrenergic agonist, has been used as an adjunct to TIVA in spine surgery. Dexmedetomidine is an effective and well-tolerated method to reduce the amount of blood loss and helps maintain hemodynamic stability during spine surgery.^12,23^ However, the administration of dexmedetomidine was associated with a decrease in MEP amplitude^18,24,25^ and did not improve pain management after the surgery.^26^ In contrast, Jiang et al^27^ showed that dexmedetomidine might increase the rate of false positive teMEPs signals and the incidence of intraoperative hypertension. However, Mishra et al^10^ received that desflurane-dexmedetomidine did not hinder MEP during spine surgery.

Neuropathic pain is caused by a lesion or dysfunction of the nervous system and is initiated by several cellular and molecular mechanisms. Compression of neural and neurovascular structures may result in neuropathic pain. Nerve injury is reported to evoke spontaneous discharges from the cell bodies of myelinated fibers at the dorsal root ganglion (DRG) cell level. The mechanism of spontaneous activity is hypothesized to be secondary to an increase in the concentrations of sodium channels in areas affected by neural microinjuries, neuromas, DRGs, and regions of demyelination.^28,29^

The use of antiepileptic drugs in the treatment of neuropathic pain is based on several similarities in the pathophysiological and biochemical mechanisms underlying neuropathic pain and epilepsy. Oral gabapentin or pregabalin given preoperatively improves pain control and reduces the opioid opioid-related side effects^30–32^ and does not interfere with spinal cord monitoring.^31^ Anderson et al^33^ randomized 50 patients to receive preoperative and postoperative administration of gabapentin as part of a multimodal pain management protocol that significantly decreased opioid use and visual analog pain scales in the first 2 postoperative days after surgery. Also, Li et al^30^ evaluated that the addition of gabapentin to intrathecal morphine resulted in reduced oral opioid consumption (0.798 vs. 1.036 mg/kg, *P*<0.015), more consistent postoperative pain scores (2.4 vs. 3.7; *P*=0.026) and a lower rate of nausea/vomiting (52% vs. 84%, *P*=0.032) and pruritus (44% vs. 72%, *P*=0.45). In contrast, Mayell et al^34^ concluded that a single preoperative dose of 600 mg gabapentin did not show a significant difference in opioid consumption or pain scores in adolescents.

In several studies, perioperative corticosteroids reduce pain and inflammation in various surgical operations, including spine surgery. The short course of postoperative steroids, adding 1 intraoperative dose and 3 postoperative doses (8 mg/dose), is associated with decreased opioid use and does not increase wound complications.^35,44^ Corticosteroids inhibit the synthesis and release of proinflammatory and anti-inflammatory mediators, resulting in a robust anti-inflammatory response. Dexamethasone suppresses the release of interleukins 1, 2, and 6 and the production of prostaglandins, reducing impulse transmission in C-type fibers, among other things. In addition, Wakamiya et al^36^ revealed that intravenous dexamethasone 0.15 mg/kg at induction of anesthesia reduces PONV (62.5% vs. 84.0%; *P*=0.02) compared with saline.

Another pain management strategy that has become popular in this patient population over the past few years is intravenous methadone. The use of methadone can provide analgesia for up to 72 hours after complex spinal surgery and is not associated with increased side effects compared with intravenous drug administration.^22,37,38^ However, Shaw et al^39^ did not show decreased opioid usage when methadone was used for pain management after scoliosis surgery. In addition, Sadhasivam et al^40^ showed that several low doses of methadone (blood methadone levels <100 ng/mL) did not lead to toxic concentrations of methadone, provided practical and lasting pain relief without respiratory depression and QT interval prolongation in children undergoing painful surgery, and against the background of complex analgesia continuous use of methadone low analgesic concentrations were used.

### Regional Anesthesia in Pain Management

Also, over the years, regional anesthesia gained popularity as an adjunct for multimodal pain management in scoliosis surgery. Table 3 summarizes the literature describing the influence of regional anesthesia on pain management in patients undergoing scoliosis surgery. Intrathecal morphine is often added to general anesthesia to prevent pain after major surgeries. Intrathecal morphine provides safe and effective postoperative analgesia, even in patients with spinal cord syrinx.^45^ Nevertheless, Thompson et al,^46^ in their retrospective study, represented 25 years of experience with intrathecal morphine and showed that patients receiving intrathecal morphine had lower pain scores (*P*=0.001) and longer time to first opioid (*P*=0.001). However, there was an increased frequency of respiratory depression (2% vs. 0%), pruritus (6% vs. 2%), nausea, and vomiting (22% vs. 9%). These side effects limit the routine use of intrathecal morphine for pain management in spine surgery.

Epidural analgesia with local anesthetics and/or opioids is frequently performed after major surgical procedures. Continuous analgesia through 1 or 2 epidural catheters placed by the surgeon at the end of the procedure has provided efficient postoperative pain control after the scoliosis correction.^47,48^ In contrast, Cohen et al^49^ did not detect any consistent difference in pain management when comparing epidural anesthesia with a placebo. However, Adeyemo et al^50^ received that epidural patient-controlled analgesia provided optimal pain control (*P*<0.05) compared with intravenous patient-controlled analgesia. In addition, epidural catheters are effective in pain management after spine surgery. Unfortunately, high failure rates and complications after epidural catheters like hematoma and epidural or vascular puncture are rare but may have profound implications. Ultrasound-guided peripheral nerve blocks reduce these risks and thus should be favored, especially in significant spine surgeries.

### Author’s Preferred Treatment

The new regional nerve block, the ultrasound-guided erector spinae plane block (ESPB), has recently gained popularity.^51–53^ ESPB has a better safety profile than neuraxial analgesia. Vergari et al^54^ received that bilateral ultrasound-guided ESPB improved postoperative analgesia, reduced the NRS score (1.7 vs. 6.3; *P*<0.001), and reduced opioid consumption (10 vs. 17; *P*<0.001). One disadvantage of this block is the short duration of action in a single injection. However, dexamethasone given an hour before the regional nerve block can prolong the time of a nerve block to 48 hours.^55^

## CONCLUSION

There is no consensus on optimal pain management for patients undergoing spine surgery. However, ensuring adequate postoperative pain control while minimizing opioids is essential in anesthesia for spine surgery. Therefore, TIVA with remifentanil and propofol should be a gold standard in major spine procedures to reduce the influence on SSEP monitoring. Also, combining safe and effective regional anesthesia, predominantly peripheral nerve blocks like erector spinae plane block, with multimodal analgesia, especially dexamethasone, should be a priority in spine surgery.
